# Australian mosquito assemblages vary between ground and sub-canopy habitats

**DOI:** 10.1186/s13071-021-04999-6

**Published:** 2021-10-07

**Authors:** Boni F. Sebayang, Tanya L. Russell, Kyran M. Staunton, Michael Townsend, Christopher Paton, Tovi Lehmann, Thomas R. Burkot

**Affiliations:** 1 Australian Institute of Tropical Health and Medicine (AITHM), James Cook University, Cairns, QLD 4878 Australia; 2 Laboratory of Malaria and Vector Research, National Institute of Allergy and Infectious Diseases (NIAH), National Institutes of Health (NIH), Rockville, MD USA

**Keywords:** Vertical distribution, Australian mosquitoes, Mosquito traps, Height

## Abstract

**Background:**

The surveillance and control of mosquito-borne diseases is dependent upon understanding the bionomics and distribution of the vectors. Most studies of mosquito assemblages describe species abundance, richness and composition close to the ground defined often by only one sampling method. In this study, we assessed Australian mosquito species near the ground and in the sub-canopy using two traps baited with a variety of lures.

**Methods:**

Mosquitoes were sampled using a 4 × 4 Latin square design at the Cattana Wetlands, Australia from February to April 2020, using passive box traps with octenol and carbon dioxide and three variations of a sticky net trap (unbaited, and baited with octenol or octenol and carbon dioxide). The traps were deployed at two different heights: ground level (≤ 1 m above the ground) and sub-canopy level (6 m above the ground).

**Results:**

In total, 27 mosquito species were identified across the ground and sub-canopy levels from the different traps. The abundance of mosquitoes at the ground level was twofold greater than at the sub-canopy level. While the species richness at ground and sub-canopy levels was not significantly different, species abundance varied by the collection height.

**Conclusions:**

The composition of mosquito population assemblages was correlated with the trap types and heights at which they were deployed. *Coquillettidia* species, which prefer feeding on birds, were mainly found in the sub-canopy whereas *Anopheles farauti*,* Aedes vigilax* and *Mansonia uniformis*, which have a preference for feeding on large mammals, were predominantly found near the ground. In addition to trap height, environmental factors and mosquito bionomic characteristics (e.g. larval habitat, resting behaviour and host blood preferences) may explain the vertical distribution of mosquitoes. This information is useful to better understand how vectors may acquire and transmit pathogens to hosts living at different heights.

**Graphical abstract:**

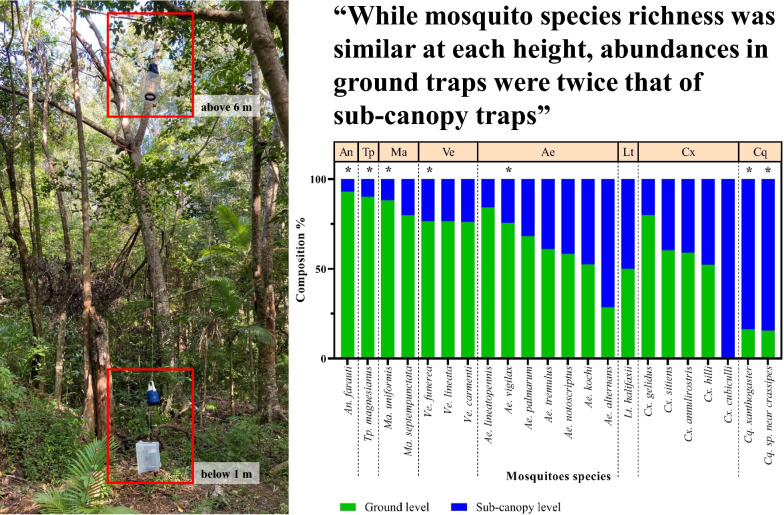

## Background

Mosquitoes are competent vectors for arboviruses, helminths and protozoans that can affect animal and human health [1]. Nearly half of the human population is at risk of mosquito-borne diseases [1]. The geographic risk of exposure to mosquito-borne diseases is increasing as vector distributions change, driven by a variety of factors, including climate change and the movement of wildlife, humans and commercial goods [1, 2]. Additionally, the risk of exposure to biting mosquitoes can change due to behavioural plasticity, frequently in response to mosquito control efforts [3, 4].

Vector control is the most effective method to control the transmission of mosquito-borne diseases [1, 4, 5]. Effective vector control requires a detailed understanding of the bionomics and distribution of mosquitos [6–8]. Mosquito distributions are highly heterogeneous, and this heterogeneity needs to be considered when designing mosquito sampling strategies [9, 10]. Gillies and Wilkes [11] categorised mosquito species as: (i) mosquitoes that only fly near the ground (< 1 m); (ii) mosquitoes that fly predominantly within 2–4 m of the ground; and (iii) mosquitoes that fly ≥ 6 m above the ground, with flight patterns hypothesised to be associated with the vertical distribution of a mosquito’s preferred blood meal hosts [11]. Clearly, the height at which a mosquito trap is placed can influence the catch rates; however, most studies only focus on quantifying the abundance and horizontal distribution of mosquitoes at the ground level [12, 13]. Recently, the extent of aerial distribution of mosquitoes was highlighted by studies of anophelines in Africa at heights up to 290 m [14], thereby demonstrating how important altitude can be to mosquito bionomics, distributions and dispersal.

Moreover, vegetation structure and meteorological conditions can influence mosquito movement and their distributions [15, 16]. The visual environment can create a physical barrier that influences the behaviour patterns of mosquito species [16]. Light intensity also impacts mosquito blood-seeking and resting behaviours and thus the distribution of mosquitoes, particularly those that are active for the entire day [15].

Mosquito assemblages can be described by their species’ abundance, richness and composition (the latter two being measures of diversity) [11, 17, 18]. Abundance or density relates to the direct count of the individuals of a species per unit of space, and richness is the count of the number of different species present, with assemblage composition reflecting the relative abundance of each species sampled [19]. Understanding these three components of mosquito populations is a fundamental step towards effectively controlling mosquito populations and any pathogens that they might transmit [13, 14, 20].

Defining mosquito abundance, richness and composition requires appropriate sampling methods [21, 22] as each sampling method has unique attributes and biases [10, 21, 23]. For example, among the commonly used methods for sampling adult mosquitoes, human landing catches (HLC) target anthropophagic species, CDC-light traps capture nocturnally active mosquitoes while barrier screens target resting behaviours of mosquitoes of all physiological states, including host-seeking mosquitoes [23–26]. Hence, studies of mosquito distributions require multiple sampling methods to comprehensively sample all species of all physiological states. In the present study, we assessed Australian mosquito species at two heights using two traps baited with a variety of lures to define species abundance, species richness and assemblage composition to define mosquito species aggregations.

## Methods

### Study site and study period

The study was conducted in the Cattana Wetlands (16°49′50.027″S, 145°42′18.611″E), Queensland, Australia between February and April 2020 under a permit issued by the Cairns Regional Council. This 80-ha environmental park encompasses palustrine and riverine ecosystems with rainforest trees, including *Archontophoenix alexandraei*, *Melaleuca* spp., *Corymbia* spp., *Ficus* spp. and *Pandanus* spp., as well as wetland grasses and sedges characterised by Para Grass and Navua Sedge [27]. The Cattana Wetlands provides habitat for a range of ground and arboreal amphibians, reptiles, mammals and birdlife.

### Sample stations

Mosquitoes were sampled concurrently within the Cattana Wetlands at each of four stations using traps set at two heights: ground level (within 1 m of the ground) and in the sub-canopy (6 m above the ground). The sub-canopy layer at the stations is characterised by juvenile trees, shrubs and herbs growing under the main canopy layer [28]. The sub-canopy normally is defined as the layer from 5 m above the ground and extending to the first branches of the main canopy [28]. The minimum distance between sampling stations was 100 m to minimise interactions between traps at different stations. Each sampling station had a tree with a branch at least 6 m above the ground that was capable of supporting a weight > 5 kg (combined maximum weight of a mosquito trap with lure), was accessible by walking and was not exposed to direct sunlight. Wind speed, temperature and humidity were recorded using a weather meter (Kestrel AU, East Melbourne, VIC, Australia) at each height level. Individuals servicing traps applied DEET-based mosquito repellent before visiting trapping stations (Aerogard; Reckitt Benckiser, Sydney, Australia).

### Mosquito traps

**Fig. 1:**
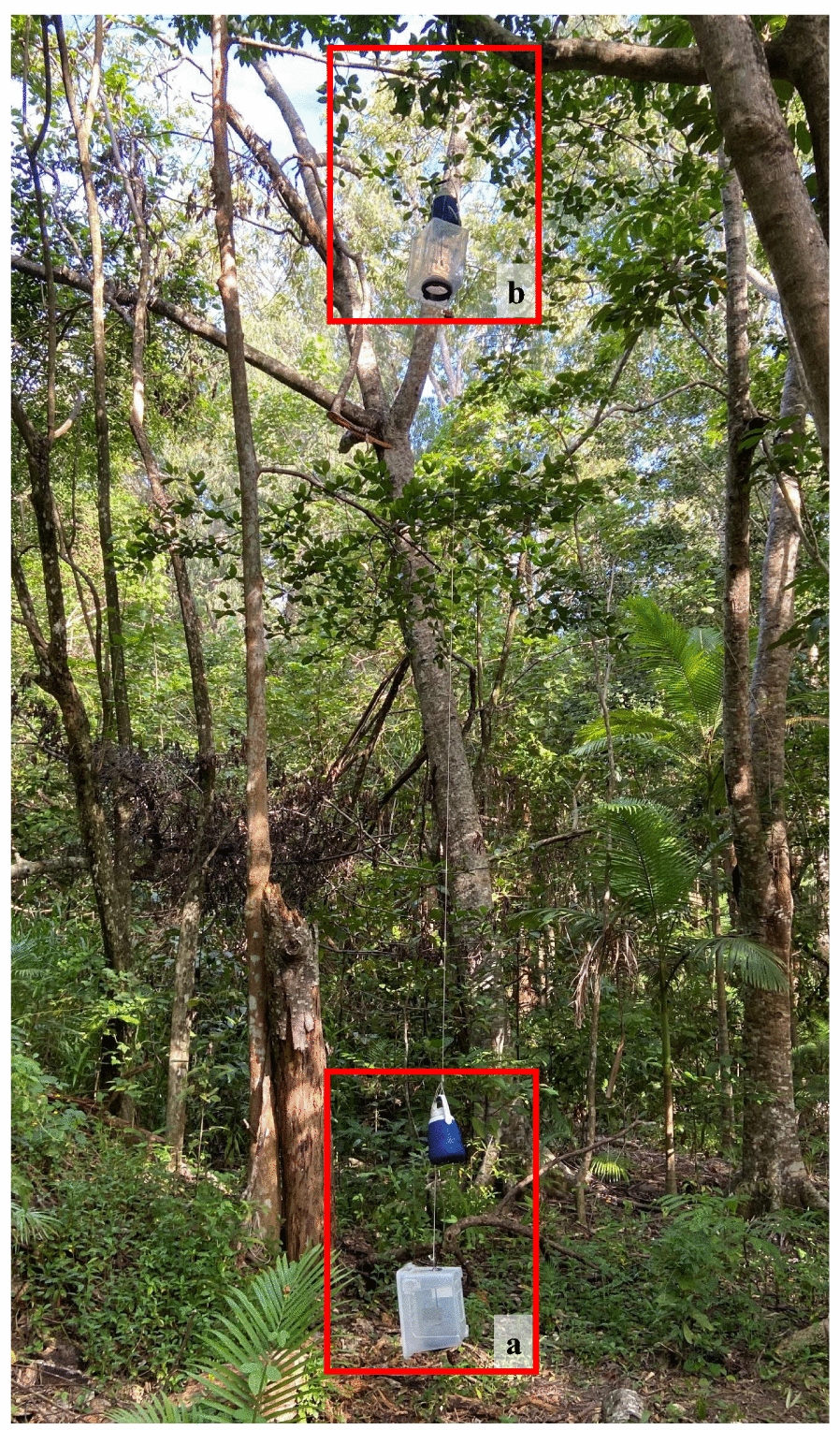
Passive box trap with octenol and dry ice (POD) as lures at ground level (< 1 m) (**a**) and sub-canopy level (> 6 m) (**b**)

**Fig. 2:**
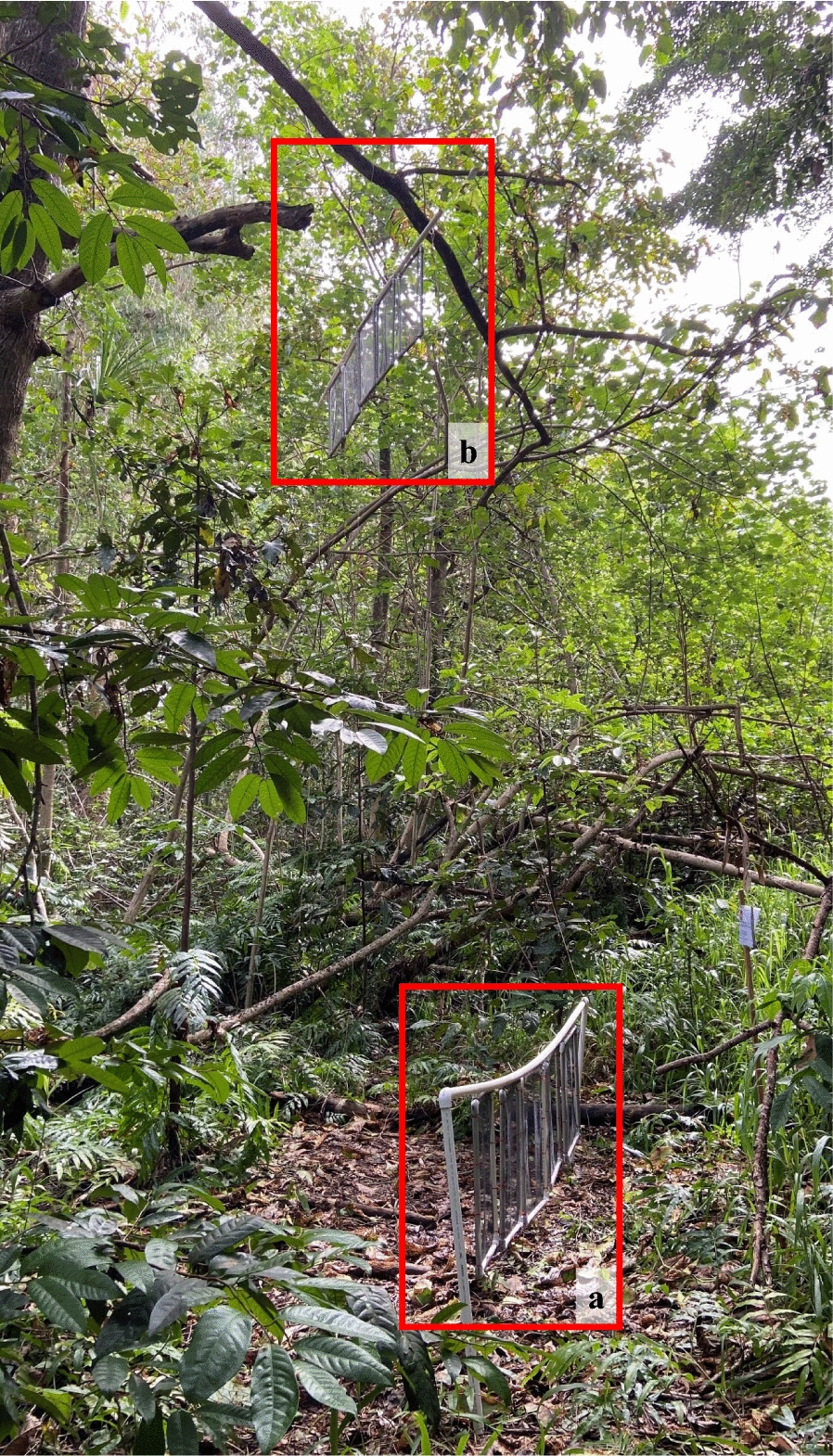
Sticky net traps without any lure (S) at ground level (< 1 m) (**a**) and at sub-canopy level (> 6 m) (**b**)

Two types of traps were used for sampling mosquitoes: the sticky net trap [14] and the passive box trap (POD) [29, 30]. The PODs were constructed of clear 37 × 29 × 27-cm (20 L) polyethylene boxes, with mosquitoes entering the trap through an inverted mesh funnel with an external opening of 616 cm^2^, tapering to 78.5 cm^2^ inside the trap (Fig. 1). The PODs were baited with 1-octen-3-ol (octenol) (Mozzie Attract; The Kelly Company Ply Ltd., Seven Hills, Australia) and 1.5 kg of dry ice in an insulated cooler from which carbon dioxide (CO_2_) was emitted. The sticky net traps (Fig. 2) were 40 × 60-cm rectangular frames constructed with 25-mm-diameter PVC pipe on which black polyester netting (36 holes per cm^2^ mesh) with a film of 60 g/m^2^ adhesive (TAD™ All weather; Trece Adhesive Division™, Grand Rapids, MI, USA) was applied. Sticky net traps were used either without any lures (S), baited with octenol (SO) or with a combination of octenol and 1.5 kg of dry ice (SOD). Whereas SO and SOD traps consisted of netting with adhesive film on a single frame, the unbaited S traps were composed of five net frames with adhesive as a low catch rate was anticipated. Hence, four trap types were compared: the POD, S, SO and SOD.

### Experimental design

Mosquito sampling with the four trap types occurred over 16 nights using a 4 × 4 Latin square design [31]. On any given night, at each station, one of the trap types was deployed in pairs, concurrently at ground and sub-canopy levels. The trap types were allocated randomly across the four sampling stations over 4 sequential nights (full rotation) to minimise location bias. Mosquito sampling occurred between 16:00 and 08:00 hours. Traps were removed from the sampling stations every morning and transferred to the laboratory. Mosquitoes were removed from sticky net traps with fine forceps and immediately identified. Other insects were removed to “clean” the sticky net traps between each sampling effort. Mosquitoes captured by the POD were killed by placing them in a freezer for at least 1 h and then transferred to an airtight container at − 20 °C until identified. All mosquitoes were identified morphologically under a stereomicroscope (Olympus, Tokyo, Japan) to species and sex [32], and the abdominal status of females classified as unfed, blood-fed or gravid. The numbers of mosquitoes, by species, sex and abdominal status, from each trap, height and station were recorded.

### Statistical analysis

The number of adult mosquitoes captured during each sampling effort was electronically recorded using the Ona platform (https://ona.io), and the final dataset was exported to MS Excel (Microsoft Corp., Redmond, WA, USA) for statistical analyses with the R statistical environment (ver. 4.0.3; https://www.r-project.org). The outcomes examined were mosquito abundance, richness and assemblage composition. Abundance was the number of specimens; richness was a count of the number of different species; and assemblage composition was the number of mosquitoes and their relative numbers. Trap ‘type’ and ‘height’ and the interaction of both parameters were used to estimate mosquito abundance and richness in separate generalised linear models (GLMs). Initial models were run using Poisson distributions, but there were overdispersed and therefore rerun using negative binomial distributions with log-link functions to account for overdispersion. An ordination of the entire female mosquito assemblage sampled was displayed graphically using non-metric multidimensional scaling (NMDS) run with the *vegan* package (ver. 2.5–7) [33]. In addition, the *tidyverse* package (ver. 1.3.0) was used to display the accumulated species richness curves of mosquitoes against the 16 nights sampling effort of each trap type by height. Lastly, the influence of both trap type and height, as well as their interaction, on assemblage composition and the individual species were also analysed using the likelihood ratio test (LRTs) in the *mvabund* package (ver. 3.6.11) [34]. LRTs compared mosquito composition, either including or not including the interaction between trap types and height.

## Results

### Mosquitoes captured

A total of 46,474 mosquitoes (137 males and 46,337 females, 12 which were engorged, but not any gravid mosquitoes) were captured over the 16 nights of collecting, from nine genera [*Culex* (*n* = 18,889), *Aedes* (*n* = 14,117), *Verralina* (*n* = 7,233), *Anopheles* (*n* = 4581), *Coquillettidia* (*n* = 825), *Mansonia* (*n* = 640), *Tripteriodes* (*n* = 70), *Lutzia* (*n* = 16) and *Uranotaenia* (*n* = 1)]. Of these 46,474 mosquitoes, 102 specimens were not identifiable due to their poor condition (Table 1).

**Table 1:** Total numbers of male and female mosquitoes captured in Cattana Wetlands, Australia, by genera

Genera	Male (*n*)	Female (*n*)		Total (*n*)
		Fed	Unfed	
*Aedes*	20	3	14,094	14,117
*Anopheles*	0	0	4581	4581
*Coquillettidia*	0	0	825	825
*Culex*	43	5	18,841	18,889
*Lutzia*	0	0	16	16
*Mansonia*	0	0	640	640
*Tripteroides*	0	0	70	70
*Uranotaenia*	0	0	1	1
*Verrallina*	0	4	7229	7233
Unidentified	74	0	28	102
Total	137	12	46,325	46,474

A total of 27 mosquito species were identified (Table 2), of which the seven most abundant species, comprising 92.3% of all specimens captured, were: *Culex annulirostris* (*n* = 13,787; 30.1%), *Aedes vigilax* (*n* = 7986; 17.5%), *Aedes kochi* (*n* = 5312; 11.6%), *Anopheles farauti* (n = 4577; 10.0%). *Verrallina funerea* (*n* = 4196; 9.2%), *Culex sitiens* (*n* = 4054; 8.9%) and *Verrallina carmenti* (*n* = 2300; 5.0%). Twenty species comprised the remaining 7.7% (*n* = 3532) of mosquitoes sampled.

**Table 2:** Total numbers of identified female mosquitoes captured in Cattana Wetlands, Australia, by species

Trap types	POD (*n*)		SOD (*n*)		SO (*n*)		S (*n*)		Total
Mosquito species	Ground level	Sub-canopy level	Ground level	Sub-canopy level	Ground level	Sub-canopy level	Ground level	Sub-canopy level	
*Aedes alternans* (Westwood)	24	60	0	0	0	0	0	0	84
*Aedes kochi* (Donitz)	2355	2261	436	254	2	2	2	0	5312
*Aedes lineatopennis* (Ludlow)	16	3	0	0	0	0	0	0	19
*Aedes notoscriptus* (Skuse)	233	162	6	8	0	1	0	1	411
*Aedes palmarum* (Edwards)	30	11	0	3	0	0	0	0	44
*Aedes tremulus* (Theobald)	38	25	0	1	0	0	0	0	64
*Aedes vigilax* (Skuse)	5962	1933	57	23	8	1	2	0	7986
*Aedes vittiger* (Skuse)	0	1	0	0	0	0	0	0	1
*Anopheles amicatus* (Edwards)	1	0	0	0	0	0	0	0	1
*Anopheles brancroftii* (Giles)	3	0	0	0	0	0	0	0	3
*Anopheles farauti* (Laveran)	3963	266	292	55	1	0	0	0	4577
*Coquillettidia sp. near crassipes* (Marks)	101	545	0	0	0	0	0	0	646
*Coquillettidia xanthogaster* (Edwards)	29	150	0	0	0	0	0	0	179
*Culex annulirostris* (Skus)	8117	5663	0	7	0	0	0	0	13,787
*Culex cubiculli* (Marks)	0	9	0	0	0	0	0	0	9
*Culex gelidus* (Theobald)	398	100	0	0	0	0	0	0	498
*Culex hilli* (Edwards)	65	59	0	0	0	0	0	0	124
*Culex pullus* (Theobald)	0	2	0	0	0	0	0	0	2
*Culex sitiens* (Weidemann)	2445	1609	0	0	0	0	0	0	4054
*Lutzia halifaxii* (Theobald)	8	8	0	0	0	0	0	0	16
*Mansonia septempunctata* (Theobald)	146	37	0	0	0	0	0	0	183
*Mansonia uniformis* (Theobald)	403	54	0	0	0	0	0	0	457
*Tripteroides magnesianus* (Theobald)	62	7	0	0	0	0	1	0	70
*Uranotaenia albescens* (Theobald)	0	0	0	0	0	0	1	0	1
*Verrallina carmenti* (Taylor)	1747	551	0	2	0	0	0	0	2300
*Verrallina funerea* (Theobald)	3177	983	23	3	4	1	5	0	4196
*Verrallina lineata* (Taylor)	551	166	0	2	0	0	0	1	720
Total	29,874	14,665	814	358	15	5	11	2	45,744

Ground level: within 1 m of the ground (< 1 m); sub-canopy level: 6 m above the ground (≥ 6 m)

Most female mosquitoes were collected from PODs (total = 44,905, 96.9%) and only 3.1% were collected from the variants of sticky net traps (Table 2). This trend was consistent for the seven main mosquito species, with the exception of *Cx. annulirostris* and *Cx. sitiens*. The PODs had a higher efficiency, capturing more species (total of 26 species) than the variants of sticky net traps, which only captured 12 out of 27 species (Table 3). *Uranotaenia albescens* (*n* = 1) was caught only in the unbaited sticky net trap (trap S) and not by the POD.

**Table 3:** The total abundance and species richness of identified female mosquitoes captured, by trap types and height

Trap types^a^	Abundance of species^b^			Species richness^b^		
	Height		Grand total	Height		Grand total
	Ground level	Sub-canopy level		Ground level	Sub-canopy level	
S	11	2	13	5	2	7
SO	15	5	20	5	5	7
SOD	814	358	1172	8	8	11
POD	29,874	14,665	44,539	23	23	26
Grand total	30,714	15,030	45,744	24	24	27

^a^POD, Passive box trap with octenol and dry ice; S, sticky net trap without any lure; SO, sticky net trap with octenol; SOD, sticky net trap with octenol and dry ice

^b^Abundance is the number of specimens collected; richness is the number of different species collected

The average (± standard deviation) nighttime temperatures at the ground and sub-canopy levels were 26.3 ± 0.07 °C and 25.4 ± 0.07 °C, respectively, with an average humidity of 85.2 ± 0.17 and 82.0 ± 0.30%, respectively. The average wind speed at ground level was 0.26 ± 0.03 mph, and 0.32 ± 0.04 mph at sub-canopy level. Limited variability in the temperature, relative humidity and wind speed throughout the study precluded analysis for associations with trap catches and, consequently, these parameters aware not included in the statistical models.

### Influence of trap and height on mosquito relative abundance and species richness

**Fig. 3:**
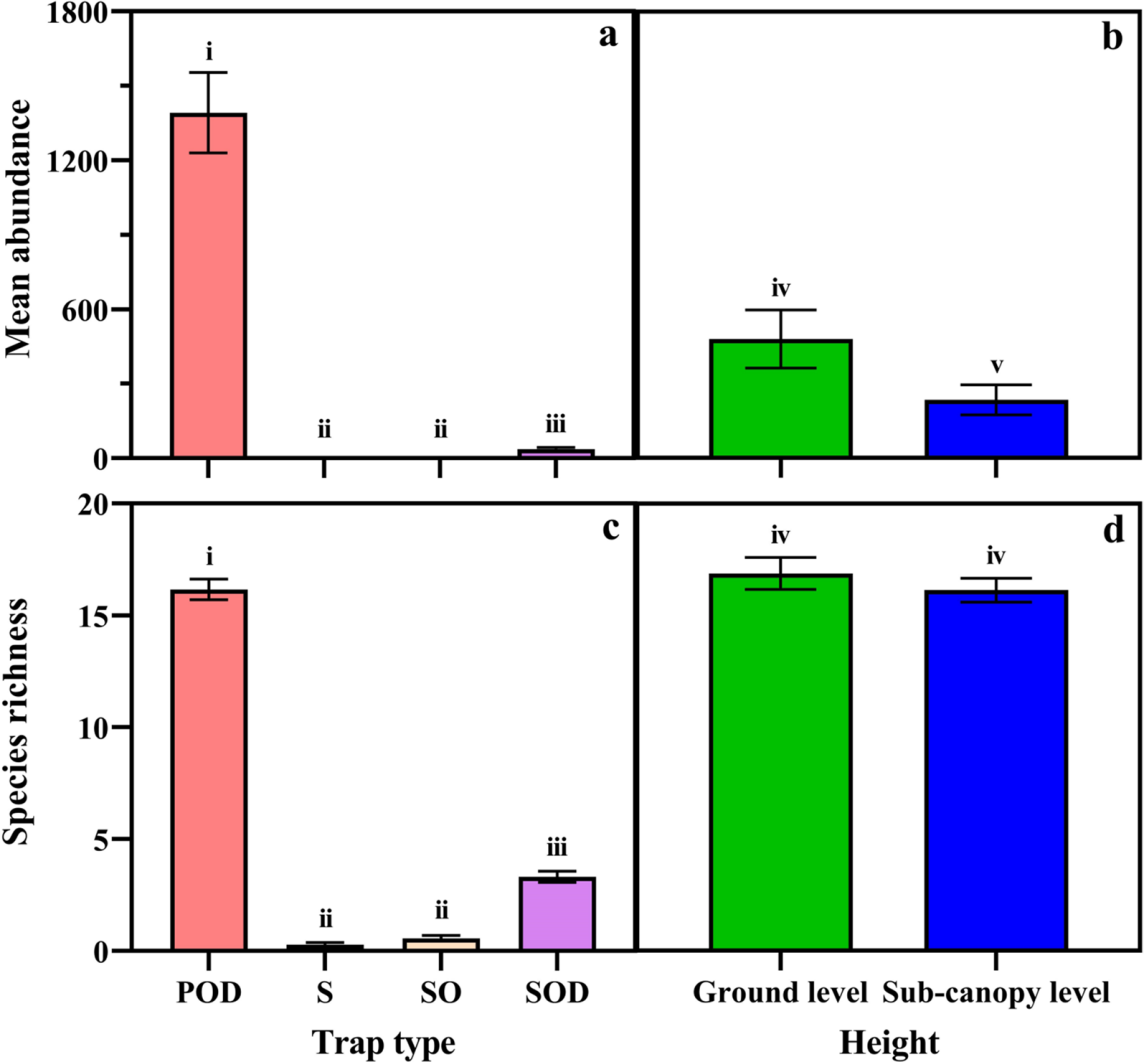
The mean nightly/daily abundance and species richness of mosquitoes captured by different trap types at two heights (ground level and . **a** Mosquito abundance by trap type, **b** mosquito abundance by height, **c** species richness by trap type, **d** species richness by height. Note: Data are presented as the means ± standard deviation (SD) per night. Abbreviations: POD, Passive box trap with octenol and dry ice; S, sticky net trap without any lure; SO, sticky net trap with octenol; SOD, sticky net trap with octenol and dry ice

Mosquito abundance was significantly influenced by trap type (GLM, *P* < 0.001; Table 4; Fig. 3a). PODs caught significantly (1391.7 ± 161.8; mean ± SD) more mosquitoes than any sticky net trap. Among the sticky net traps, significantly more mosquitoes were captured when octenol and dry ice were used (36.6 ± 6.9) than with either the sticky net trap with octenol (0.63 ± 0.15) or the sticky net trap without any lure (0.4 ± 0.1) (Fig. 3a).

**Table 4:** Influence of trap and height on mosquito relative abundance and species richness

Factors	Abundance			Species richness		
	*χ*^2^	*df*	*P* value	*χ*^2^	*df*	*P* value
Trap type	1400.02	3	< 0.001	974.85	3	< 0.001
Height	23.62	1	< 0.001	1.58	1	0.209
Trap type × Height	1.45	3	0.694	5.89	3	0.117

Two separate generalised linear models with negative binomial distributions were used to estimate the influence of experimental factors for both mosquito abundance (*R*^2^ = 0.922) and species richness (*R*^2^ = 0.921)

The average (± standard deviation) mosquito abundance per trap-night was significantly influenced by trap height (GLM, *P* < 0.001; Table 4, Fig. 3b) with more mosquitoes captured at ground level (479.8 ± 116.9) than in the sub-canopy (234.8 ± 60.4). There was no significant interaction (GLM, *P* = 0.694) between trap type and height.

Mosquito species richness was significantly influenced by trap type (GLM, *P* < 0.001; Table 4; Fig. 3c). PODs caught significantly (16.2 ± 0.5) more species than any of the sticky net traps. Among sticky net trap types, a significantly greater richness was captured using octenol and dry ice (3.3 ± 0.3) than either the sticky net trap with octenol (0.6 ± 0.1) or the sticky net trap without any lure (0.3 ± 0.1).

Mosquito species richness was not significantly influenced by trap height (GLM, *P* = 0.209; Table 4; Fig. 3d). The mean of daily species richness estimated from all four trap types over the 16 days at ground level (17.1 ± 2.8) was not significantly different to that in the sub-canopy (16.2 ± 2.1). Lastly, species richness was not significantly influenced (GLM, *P* = 0.117) by an interaction between trap type and height.

**Fig. 4:**
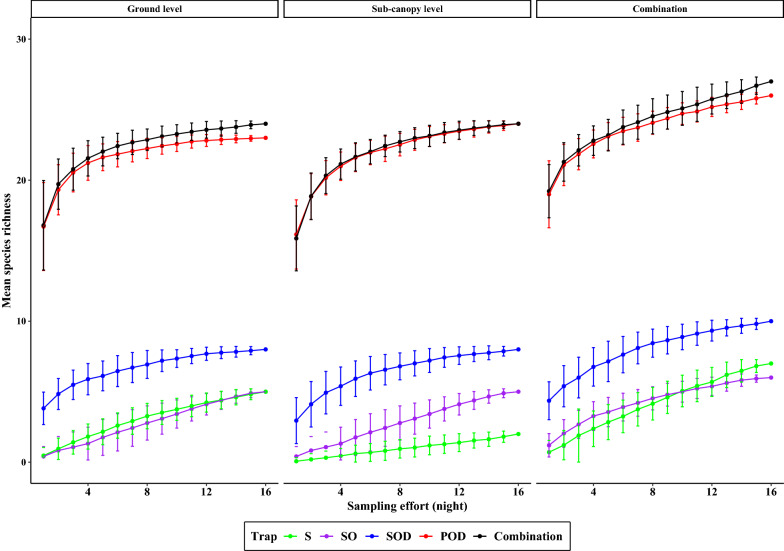
Cumulative mean species richness of mosquitoes by trap types and height per sampling effort (night) in Cattana Wetlands, Cairns, Australia. Note: data are presented as nightly cumulative means ±  SD

The mosquito species richness increased with increases in the number of sampling nights (Fig. 4). After 1 night of sampling, traps collected about 70% of the cumulative number of species collected over 16 nights of sampling (POD: 19 ± 2.37; SOD: 4.36 ± 1.34; SO: 1.2 ± 0.83; S: 0.71 ± 0.83).

### Mosquito assemblage composition

**Fig. 5:**
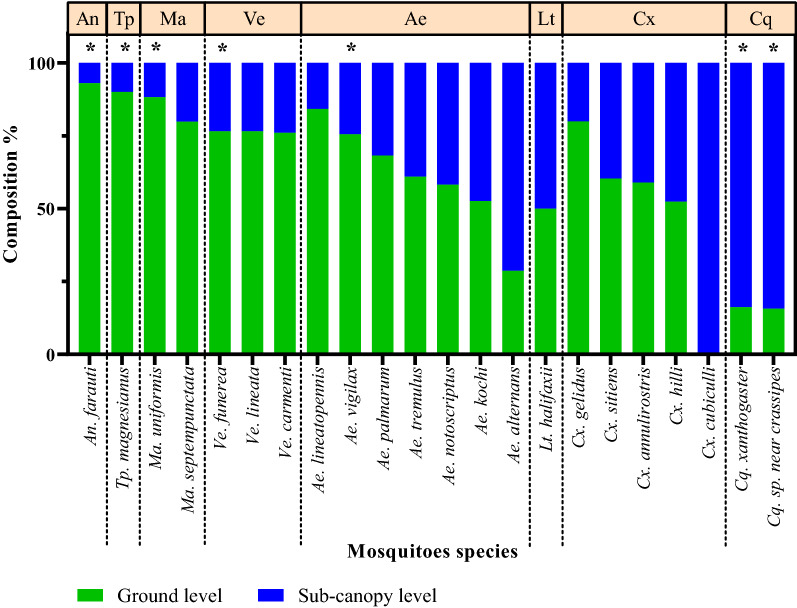
Relative composition of the 22 most abundantly sampled mosquito species by height in Cattana Wetlands, Cairns, Australia. Species whose abundance was significantly influenced by height are denoted with an asterisk. The graph excludes 5 mosquito species because the total number of each species captured was < 5. Abbreviations: *An*., *Anopheles*; *Ma*., *Mansonia*; *Ve*., *Verralina*; *Ae*., *Aedes*; *Lt.*, *Lutzia*; *Cx*., *Culex*; *Cq*., *Coquillettidia*

Variations in mosquito assemblage composition were positively correlated with trap types (manyglm function, LRT = 2276.6, *P* = 0.001), with height at which traps were deployed (manyglm, LRT = 194, *P* = 0.001) and across trap types and height (manyglm, LRT = 48.7, *P* = 0.001). The mean abundance of mosquito species (for the 22 species for which > 5 specimens were sampled) was significantly influenced by trap type (*P* = 0.001; Table 2). Additionally, mean abundances of seven mosquito species were significantly influenced by trap height (Table 5), with the mean abundances of *Ae. vigilax* (manyglm, *P* = 0.001), *An. farauti* (manyglm, *P* = 0.001), *Mansonia uniformis* (manyglm, *P* = 0.007), *Tripteroides magnesianus* (manyglm, *P* = 0.001) and *Ve. funerea* (manyglm, *P* = 0.001) being significantly greater in traps at ground level (Table 2; Fig. 4), and the mean abundances of* Coquillettidia sp. near crassipes* (manyglm, *P* = 0.002) and *Coquillettidia xanthogaster* (manyglm, *P* = 0.033) being significantly greater in traps within the sub-canopy (Table 5; Fig. 5).

**Table 5:** Seven mosquito species whose abundances were influenced by trap and height

Mosquito species	Trap type		Height	
	LRT	*P* value	LRT	*P* value
*Aedes vigilax* (Skuse)	193.349	0.001	21.173	0.001
*Anopheles farauti* (Laveran)	157.601	0.001	45.604	0.001
*Coquillettidia sp. near crassipes* (Marks)	128.046	0.001	17.098	0.002
*Coquillettidia xanthogaster* (Edwards)	78.166	0.001	10.112	0.033
*Mansonia uniformis* (Theobald)	98.73	0.001	13.026	0.007
*Tripteroides magnesianus* (Theobald)	66.933	0.001	24.343	0.001
*Verrallina funerea* (Theobald)	147.161	0.001	22.833	0.001

The likelihood tests (LRTs) were used to estimate the interaction between mosquito species with trap type and height

**Fig. 6:**
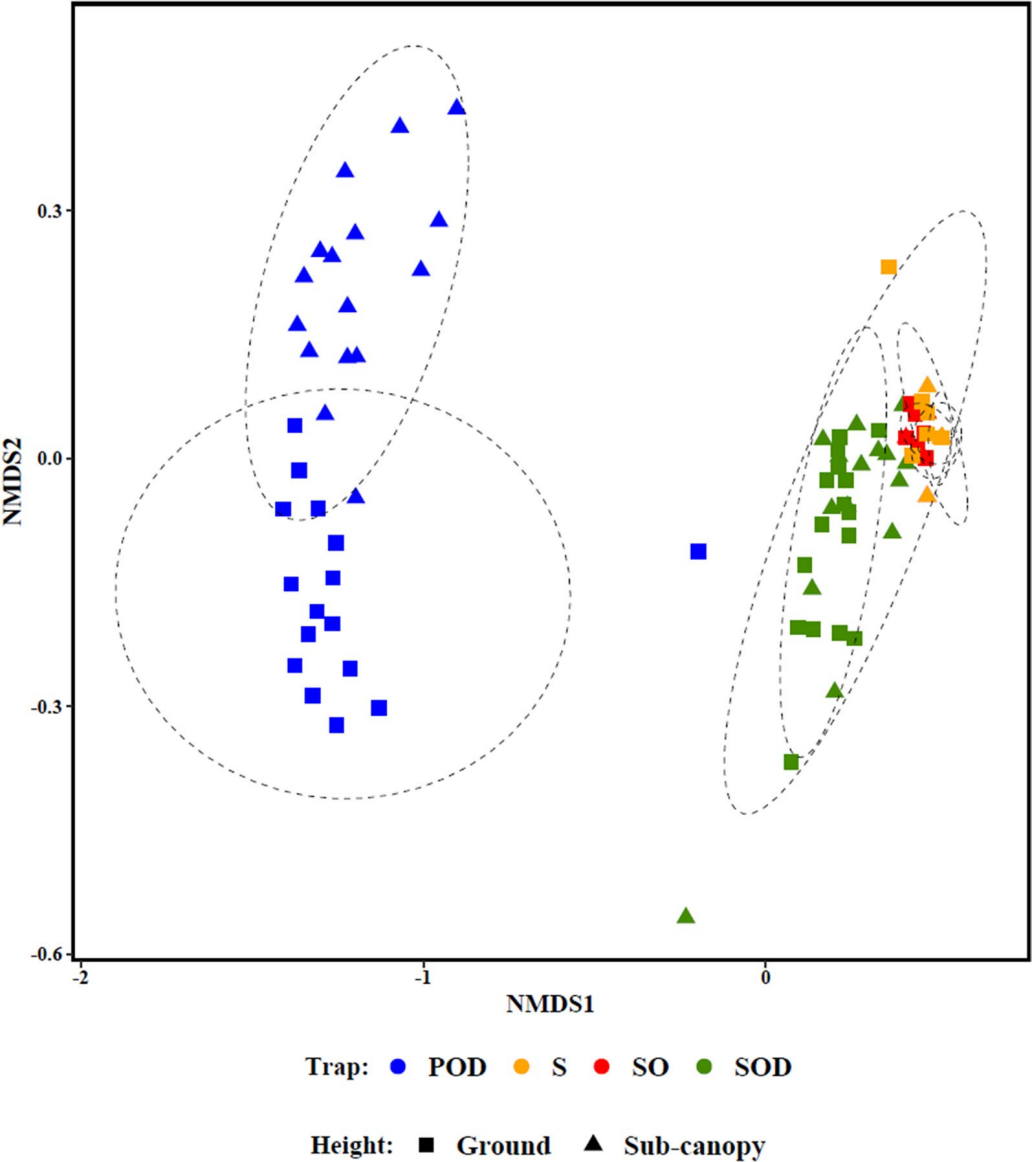
nMDS of mosquito species composition from replicated trapping efforts comparing different trap types and heights. Abbreviations: nMDS, Non-metric multidimensional scaling

A large separation between the structure of mosquito assemblages sampled by the passive and sticky net traps was seen (Fig. 6), with the assemblage captured by the POD separating into two groups by trap height. There was substantial overlap among the assemblages sampled by the sticky net trap versions. However, the assemblage captured with the SOD had a greater species richness and abundance compared to the other sticky net traps, shown as a separation between these groups in the ordination in Fig. 6.

## Discussion

This study compared the effectiveness of different traps for sampling mosquitoes and quantified the effect of trap height on mosquito assemblages. Defining trap effectiveness by the number of species captured and the abundance of each species, our results show that that the POD was significantly more effective than any of the sticky net traps. *Aedes*, *Anopheles* and *Verrallina* were the dominant mosquito genera sampled by each trap. However, while *Culex* were very abundantly sampled in PODs, very few were collected in sticky net traps, regardless of the lures used, suggesting that sticky net traps were less effective for sampling *Culex*. Despite its overall low sampling efficiency, the sticky net trap did capture one species, *Ur. albescens*, that the POD did not. Furthermore, the sticky net trap may provide information about natural flight patterns than the POD cannot as sticky traps capture mosquitoes from two horizontal directions [21]. Therefore, a combination of field sampling methods over multiple days was required to more comprehensively and representatively sample mosquito populations and thereby better describe the mosquito assemblage in an area [21, 23].

The abundance and composition of mosquito assemblages sampled clearly depended on whether traps were placed near the ground or in the sub-canopy. This is supported by previous investigations in Australia [35], The Gambia [11], Madagascar [13], Malaysia [20], Mali [14] and the USA [18]. In this study, the abundance of mosquitoes sampled at ground level was more than twofold greater than the abundance sampled in the sub-canopy. However, the richness of mosquito species was similar at both heights. Variations in abundance of mosquito species at different heights may be influenced by the structure of the vegetation the provides sugar sources [36–38], resting sites, oviposition sites or preferred blood meal hosts [39, 40]. In this study, *Ae*. *vigilax* and *Ve. funerea* were collected in significantly greater numbers at the ground level. The larvae of both of these mosquito species are found in saline and brackish water [41]. Cattana Wetlands has a number of fresh and brackish water anthropogenic lakes which may serve as larval habitats for these species and thus explain their abundance at the ground level [42, 43].

Mosquitoes with feeding preferences for large mammals are hypothesised to be predominantly found near the ground, whereas ornithophilic species may predominantly be in the sub-canopy or the canopy layer [35, 44]. For example, the ornithophilic *Culex pipiens* complex were found most frequently in the sub-canopy level where their preferred blood meal hosts are generally found [17, 18, 45]. In this study, most *Cq. sp. near crassipes* and *Cq. xanthogaster* were collected in the sub-canopy but a few were sampled at ground level, consistent with both their known ornithophilic blood-feeding habits and their preference for oviposition in freshwater wetlands with aquatic plants [32, 42]. Unsurprisingly, 94% of *An. farauti* were sampled in traps at ground level, which is consistent with their known preference for blood meals on large animals [46, 47]. While *Ae. vigilax* is known as a generalist feeder [41], this mosquito’s role as a vector of dog heartworm (*Dirofilaria immitis*) suggests a preference for biting large mammals, including dogs [32, 48]. In addition, an investigation of the host preferences of *Ma. uniformis* at Kowanyama, Australia in 1979 found that 15 out 17 blood-fed mosquitoes captured were positive for large mammals, including human, cow, pig, horse and marsupial [49, 50]. Moreover, *Cx. annulirostris* and *Cx. sitiens* were collected at both the ground and sub-canopy levels, consistent with their generalist feeding behaviours [51, 52].

## Conclusion

The POD clearly outperformed all three sticky net trap variants in both numbers and abundance of species sampled in this wetland. These results contribute new insights into mosquito communities within an Australian wetland habitat. Despite mosquitoes being overall significantly more abundant at the ground level, two species were caught in higher abundances in traps set in the sub-canopy level, with species richness comparable at ground and sub-canopy levels. This suggests that while most species more frequently inhabit lower heights in this habitat, many mosquito species also freely fly to heights of 6 m albeit in lower abundances. Potentially, variations in mosquito species distributions between these two heights are influenced by their preferred hosts’ availability, the vegetation structure, resting and larval habitats and environmental factors.

## Data Availability

The datasets supporting the conclusions of this article are available at Research Data JCU repository: https://research.jcu.edu.au/data/default/rdmp/home.

## Abbreviations

*Ae*.: *Aedes*
*An*.: *Anopheles*
CO_2_: Carbon dioxide
*Cq*.: *Coquillettidia*
*Cx*.: *Culex*
GLM: Generalised linear model
HLC: Human landing catch
LRT: Likelihood ratio
*Lt*.: *Lutzia*
*Ma*.: *Mansonia*
NMDS: Non-metric multidimensional scaling
POD: Passive box trap with octenol and dry ice
S: Sticky trap without any lure
SO: Sticky trap with octanol
SOD: Sticky trap with octenol and dry ice
*Tp*.: *Tripteroides*
*Ur*.: *Uranotaenia*
*Ve*.: *Verralina*
